# Low prevalence of diabetic retinopathy in patients with long-term type 1 diabetes and current good glycemic control - one-center retrospective assessment

**DOI:** 10.1007/s12020-021-02871-2

**Published:** 2021-09-20

**Authors:** Paulina Surowiec, Bartłomiej Matejko, Marianna Kopka, Agnieszka Filemonowicz-Skoczek, Tomasz Klupa, Katarzyna Cyganek, Bożena Romanowska-Dixon, Maciej T. Malecki

**Affiliations:** 1 Department of Metabolic Diseases, Jagiellonian University Medical College, Krakow, Poland; 2 University Hospital, Krakow, Poland; 3 Department of Ophthalmology, Jagiellonian University Medical College, Kraków, Poland

**Keywords:** Diabetic retinopathy, Type 1 diabetes, Risk factors

## Abstract

**Purpose:**

Despite progress in type 1 diabetes (T1DM) therapy, diabetic retinopathy (DR) is still a common complication. We analysed predictors and prevalence of DR in patients with T1DM lasting 10 years or more. All of the patients were considered to be currently in excellent glycemic control and treated using modern therapies.

**Methods:**

Study included 384 (80.7% women) T1DM patients participating in the Program of Comprehensive Outpatient Specialist Care at the University Hospital in Krakow between the years 2014 and 2020. A retrospective analysis of medical records was conducted.

**Results:**

The patients were on average 34 ± 9.2 years old, had a BMI 25.0 ± 3.9 and a T1DM duration of 20.5 ± 7.9 years. The mean level of HbA1c throughout the follow-up (mean duration 4.9 ± 1.4 years) was 6.9 ± 1%. The group included 238 (62.0%) patients treated with insulin pumps and 99 (25.8%) on multiple daily injections, 47 (12.2%) used both methods; almost all patients were on insulin analogues. DR was confirmed in 150 (39.1%) patients, from which 109 (28.4%) were diagnosed de novo. Severe DR was occurred in just 31 cases (8.1%). In the multivariate logistic regression, independent risk factors for the presence of DR were T1DM duration (OR 1.13; 95% CI, 1.09–1.19), HbA1c level (OR 1.41; 95% CI, 1.08–1.84), LDL level (OR 1.79; 95% CI, 1.16–2.87), and the combined presence of non-DR micro- and macrovascular chronic complications (OR 1.86; 95% CI, 1.16–3.03).

**Conclusions:**

In this highly-selected group of T1DM patients, mostly female, the prevalence of both DR at any stage and severe DR was lower than earlier reported results from other cohorts. Independent risk factors for the DR cohort did not differ from previously reported studies.

## Introduction

Diabetic retinopathy (DR) is a common chronic complication of type 1 diabetes (T1DM). It remains an important cause of vision loss and preventable blindness in adults, particularly in middle and high-income countries [1]. DR is responsible for 2.6% of global blindness [2]. In addition, other eye diseases, like glaucoma and cataracts, are more common and develop earlier in people with diabetes [3, 4]. Interestingly, it was recently reported, that the presence and degree of DR was an independent predictor of subclinical cardiovascular disease (CVD) [5–7]. A meta-analysis of 35 studies conducted world-wide in 1980–2008 estimated the overall prevalence of DR at any stage at 34.6% in a large population with a mean disease duration <8 years [8]. Moreover, the same analysis showed that people with T1DM had a three times higher risk of DR compared to people with type 2 diabetes (T2DM) (77.3% vs 25.2%) [8]. Data referring to the prevalence of DR among T1DM patients from different countries usually aggregate patients without being broken down into different treatment groups or diabetes duration [9–13]. This data is largely based on a retrospective analysis conducted in the previous decades [14, 15]. Several risk factors of DR are well-established, for example, diabetes duration, glycemic exposure, arterial hypertension and dyslipidemia [8, 16–20]. Research confirms the influence of puberty, pregnancy and diabetic kidney disease (DKD) on the occurrence and progression of DR [21–23].

Large randomised controlled studies (RCTs) showed a delay in the onset and progression of DR as well as retaining vision after the intensification of hypoglycemic treatment [24, 25]. The effect of better glycemic control lasts up to 18 years, as shown in the EDIC project [26]. RCTs also found that continuous subcutaneous insulin infusion (CSII) with insulin pump had a beneficial effect on glycemic control in adults with T1DM, compared to multiple daily injections (MDI), which may possibly result in the risk reduction of chronic complications [27]. In addition, the new methods of intensive monitoring, such as continuous glucose monitoring (CGM), have advantages over the self-monitoring of blood glucose (SMBG), and are associated with a substantial improvement of glycaemic control independently from the use of either CSII or MDI [27, 28].

There is a need to analyse the prevalence of DR at various stages in consecutive generations of T1DM patients, and to assess possible effect of use of new insulins and diabetes technologies, such as personal pumps and glucose monitoring tools. This type of research helps to define the impact of innovative therapeutic methods on DR development, identify people at high risk of this complication, plan preventive programs and estimate health care costs. While some published reports suggest that the recent progress in T1DM therapy and monitoring reduced the risk of microvascular complications, including DR [29], further evidence is needed to document this beneficial effect.

We aimed to retrospectively analyse the DR frequency and its predictors in T1DM lasting 10 years or more and in patients characterised to be currently in excellent glycemic control and treated with modern therapeutic methods over the observation period.

## Subjects and methods

A retrospective, real-world data (RWD) study of patients with T1DM participating in the Polish National Health Fund Programme of Comprehensive Outpatient Specialist Care (Polish acronym KAOS) at the University Hospital in Krakow, an academic referral centre for diabetes in southeastern Poland with a tertiary level of reference was performed. KAOS is dedicated mainly to T1DM patients, and requires regular DR screening, along with clinical consultations as well as laboratory tests done in pre-specified time frames to warrant financing the cost of each individual patient. Every patient has to be consulted by an ophthalmologist and neurologist at least once a year. Visits to the diabetologist take place regularly every three months, except for the period of pregnancy; in these cases the visits are scheduled for every month.

Each visit included checking the patient’s weight, blood pressure and collecting data on the daily insulin requirement. We included patients who remained under the care of the diabetic clinic for at least one full year between 2014–2020, and had a diagnosis of T1DM with at least a 10 year history. Paper and electronic medical records of KAOS patients in the years 2014–2020 (until May inclusive) were analysed. According to these records, 28 patients (7.3%) had only 1 ophthalmic visit, 54 (14.1%) – 2 visits, 83 (21.6%) – 3 visits, 95 (24.7%) – 4 visits, and the remaining 124 (32.3%) had 5 or more visits. For the analysis of the incidence of DR, at least 3 ophthalmic consultations were required over the study period. The following variables were collected: gender, age, weight, age at T1DM onset, type of therapy (CSII/MDI/use of metformin/other oral hypoglycemic drugs/alternative therapies, for example, DIY system), patient’s BMI, type of insulin, daily insulin requirements, other medications and comorbidities. Data, such as the patient’s weight and daily insulin requirement, was analysed on the basis of the patient’s last visit to a diabetologist’s office. Patients were assessed for the occurrence of microvascular and macrovascular chronic complications.

The annual evaluations of an ophthalmologist with dilated pupils were conducted. In case of abnormalities in the fundus image, the lesions were classified according to recommendations proposed by the American Academy of Ophthalmology [30]. The presence of diabetic macular edema and data on laser therapy or other therapeutic measures (in the past and during the analysed period) were also collected.

T1DM patients diagnosed with DR were divided into two groups, based on the stage of DR. The group of severe DR associated with a significant risk of visual loss included people with advanced non-proliferative DR that required laser therapy, intravitreal injection of anti-VEGF and proliferative DR. The second group, non-severe DR, included subjects with eye fundus lesions described as mild or moderate non-proliferative DR.

The presence of DKD was assessed on the basis of the albumin/creatinine ratio and level of eGFR according to the recommendations of Kidney Disease Improving Global Outcomes. DKD was being diagnosed in cases of history of persistent albuminuria, overt proteinuria and/or persistently reduced estimated glomerular filtration rate below 60 ml/min/1.73 m^2^ [31]. Albuminuria was defined as an albumin-to-creatinine ratio between 3 and 30 mg/mmol and overt proteinuria as an albumin-to-creatinine ratio >30 mg/mmol, in two repeated morning urine samples. Peripheral and autonomic neuropathy were diagnosed based on an annual neurological examination. Arterial hypertension was identified according to the medical history or/and use of anti-hypertensive medications. Coronary artery disease (CAD) was diagnosed if the patient had a myocardial infarction or medical record that provided the evidence of ischemic heart disease diagnosed by a cardiologist. Patients over 35 years had an ECG performed annually, which was analysed by the attending physician -if abnormalities occurred, the patients were referred to a cardiologist and further diagnostic was performed, including additional tests (ECG Holter, echocardiography). The presence of micro and macrovascular chronic complications was analysed individually. This includes microvascular complications (DKD, peripheral neuropathy, autonomic neuropathy) and macrovascular disease (CAD, myocardial infarction, stroke, transient ischemic attack and lower limb atherosclerosis diagnosed on basis of the results of the ankle-brachial index, which was measured every 2 years). We combined all complications other than DR in one variable. For the calculations, a graded classification system for variable of combined complications other than DR was used. A scale from 1 to 3 was devised—each grade represented the total number of complications other than DR, 3 complications being the maximum number observed in the examined cohort. The number of patients with grade 1 (1 complication) was 114 (29.7%), grade 2–14 (3.7%), grade 3–2 (0.5%), respectively. Information concerning pregnancy during the period of observation was also collected.

The European Society of Cardiology guidelines for the diagnosis and treatment of dyslipidemia have changed twice between 2014 and 2020. Initially, the recommendations from 2011 were enforced, then in August 2016 they were changed and the last update took place in August 2019 [32–34]. Patients, when diagnosed with dyslipidemia, were treated in accordance with the guidelines applicable in a given year, with the exception of pregnant women. The mean values of total cholesterol (TC), LDL cholesterol, HDL cholesterol and triglyceride (TG) levels during the observation period were available, information on thyroid diseases was also collected. Annual laboratory test results (HbA1c level, renal parameters, lipid profile, TSH) were gathered. The HbA1c levels were determined using analytical methods certified by the National Glycohemoglobin Standardization Program. Present and past cigarette smoking was taken into consideration.

### Statistical analysis

The normality of continuous variable distribution was assessed by the Shapiro–Wilk test. Differences between groups were analysed with Student’s test or nonparametric tests, as appropriate. The study results are presented as arithmetic means (x̄), ± standard deviations (SD) or number (N) and percentages (%). Uni- and multivariate logistic regression analysis was employed to determine the associations of the independent variables with the presence of DR, with respect to potential confounding variables. The list of variables used for univariate analysis is shown in Table 2. For each variable, an odds ratio (OR) with confidence intervals (CI) for the presence of DR were provided. For the multivariate model, significant variables from univariate analysis were included. Instead of analysing each complication separately, the variable of the presence of any micro or macrovascular chronic complication was used. LDL level was chosen as a marker of a lipid disorder. All statistical analyses were performed using R ver. 4.0.2 statistical software (http://www.r-project.org/). The results were considered significant at a significance level of *p* < 0.05.

## Results

Out of 521 patients under the care of KAOS in 2014–2020, we included data from 384 T1DM patients with at least a 10-year history of the disease. The average number of ophthalmic consultations during the study period was 3.7 + /− 1.5. The mean follow-up was 4.9 + /− 1.4 years. This group included 310 (80.7%) women. On average, this group was characterised by mean age of 34 ± 9.2 years, mean BMI 25.0 ± 3.9 kg m^–2^ and mean T1DM duration 20.5 ± 7.9 years. The average number of measurements of HbA1c during the follow-up was 2.9 + /−1.2 per year. The mean HbA1c of all patients throughout the follow-up period was 6.9 ± 1%, 227 patients (59.1%) achieved the recommended target value of HbA1c < 7% and 150 people (39.1%) achieved an average HbA1c < 6.5%. The mean daily insulin requirement/kg body weight was 0.7 ± 0.2 IU/kg. The average mean LDL level was 2.5 ± 0.6 mmol/l, and HDL level – 1.8 ± 0.4 mmol/l. The mean eGFR value was 66.3 ± 12.0 ml/min/1.73 m^2^. Detailed clinical characteristics of the group are presented in Table 1. The analysed group included 238 (62%) patients treated with CSII during the entire observation period; 99 (25.8%) were using only MDI; 47 (12.2%) used both methods during the follow-up period. All patients were using short-acting analogues [insulin lispro – 183 people (47.7%), aspart – 138 (35.9%), glulisine – 49 (12.8%), two or more short-acting analogues – 14 (3.6%)]; 144 people (98.6% MDI users) were on long-acting analogues [glargine U100 – 58 people (39.7%), glargine U300 – 36 (24.7%), detemir – 29 (19.9%), degludec – 15 (10.3%)]. More than half of the women—163 (52.6% of women, 42.4% of the entire group)—were pregnant within the observation period. Every third person [132 (34.4%)] required a cardiological consultation for various reasons, related to clinical signs and/or symptoms and/or an abnormal ECG tracing. Supplementary data on clinical characteristics are provided in the online Appendix 1 showing the use of concomitant medications.

**Table 1:** Study group characteristics according to the presence of DR

Variable	Whole study group			Presence of DR			Absence of DR			*P* value
	Mean	SD	Median	Mean	SD	Median	Mean	SD	Median	
Gender F/M [*n*/*n*]/[%/%]	[310/74]/[80.7/19.3]	-	-	[123/27]/[82/18]	-	-	[187/47]/[79.9/20.1]	-	-	0.709
Model of treatment [CSII/MDI/both] [*n*/*n*/*n*]/[%/%/%]	[238/99/47]/[62/25.8/12.2]	-	-	[90/39/21]/[60/26/14]	-	-	[148/60/26]/[63.3/25.6/11.1]	-	-	0.675
Age [yrs]	34.4	9.2	34	37.8	9.4	36	32.3	8.5	31.5	≤0.001
Diabetes duration [yrs]	20.5	7.9	20	25.0	8.4	24	17.7	6.1	16.5	≤0.001
HbA1c [%]	6.9	1.1	6.8	7.1	1	7	6.8	1.1	6.6	0.003
Daily Insulin Dose/kg [IU/kg]	0.7	0.2	0.7	0.7	0.2	0.7	0.7	0.3	0.7	0.317
BMI [kg/m^2^]	25	3.9	24.5	25.1	4	24.4	24.9	3.9	24.6	0.478
Total cholesterol level [mmol/l]	4.6	0.7	4.6	4.8	0.7	4.8	4.6	0.7	4.5	0.009
LDL level [mmol/l]	2.5	0.6	2.4	2.6	0.7	2.5	2.4	0.6	2.3	0.001
HDL level [mmol/l]	1.8	0.4	1.8	1.8	0.4	1.8	1.8	0.4	1.8	0.344
TG level [mmol/l]	0.9	0.5	0.8	1	0.5	0.8	0.9	0.4	0.8	0.243
eGFR level [ml/min/1.73 m^2^]	66.3	12	64.8	66.5	13.4	65	66.2	10.9	64.3	0.824
Hypertension [*n*][%]	[57][14.8]			[35][23.3]			[22][9.4]			≤0.001

DR was confirmed in 150 (39.1%) patients; of them, 109 (28.4%) were classified as diagnosed de novo in the analysed time period. The majority were patients diagnosed with mild non-proliferative DR - 130 (86.7% of all affected by DR). Severe DR was found in 31 people (8.1% of the entire study group and 20.7% of the DR group), including 13 people with PDR and 4 with advanced non-proliferative DR. In addition, 4 patients (1%) had DME (3 in the follow-up, 1 in the past), 30 individuals (7.8%) underwent laser therapy (2 for reasons other than DR) and 4 others (1.0%) were treated with intravitreal injections with anti-VEGF, 8 (2.1%) had cataracts, and 5 (1.3%) underwent vitrectomy.

The patients were compared according to the presence of DR. The group with DR was older (mean: 37.8 ± 9.4 vs. 32.3 ± 8.5 years; *p* ≤ 0.001), had a longer duration of diabetes (25.0 ± 8.43 vs. 17.7 ± 6.1 years; *p* ≤ 0.001), achieved a higher mean level of HbA1c (7.1 ± 1 vs. 6.8 ± 1.11%; *p* = 0.003), had higher mean LDL cholesterol level (2.6 ± 0.7 vs. 2.4 ± 0.6 mmol/l; *p* = 0.001) and mean total cholesterol (TC) level (4.8 ± 0.7 v 4.6 ± 0.7 mmol/l; *p* = 0.009). The treatment regimens (CSII/MDI/combination therapy) were used with similar frequencies. Details are presented in Table 1.

Additionally, T1DM female (*n* = 310) and male patients (*n* = 74) were compared. The men were older (mean: 34.8 ± 12.2 vs. 34.4 ± 8.4 years; *p* ≤ 0.001), had a shorter duration of diabetes (19.7 ± 7.6 vs. 20.7 ± 8 years; *p* ≤ 0.001), had higher HbA1c level (7.5 ± 1 vs. 6.8 ± 1 mmol/l; *p* = 0.003), and lower LDL cholesterol level (2.4 ± 0.6 vs. 2.5 ± 0.7 mmol/l; *p* = 0.001). The prevalence of any DR and the severity of DR were not associated with gender (*p* = 0.71; *p* = 0.15). During the study 68 (17.7%) women with T1DM and the diagnosis of DR registered in this cohort were pregnant; in 4 of these patients, during the pregnancy DR was diagnosed de novo and in 14 cases a DR progression was observed.

In univariate logistic regression, significant predictors of the presence of DR were older age (OR 1.07; 95% CI, 1.05–1.1), longer diabetes duration (OR 1.16; 95% CI, 1.12–1.2), higher HbA1c level (OR 1.26; 95% CI, 1.04–1.54), peripheral neuropathy (OR 4.72; 95% CI, 2.97–7.61), autonomic neuropathy (OR 7.76; 95% CI, 2.84–27.25), DKD (OR 6.28; 95% CI, 2.23–22.37), higher LDL level (OR 1.9; 95% CI, 1.34–2.75), arterial hypertension (OR 2.87; 95% CI, 1.62–5.2), and the presence of any micro- or macrovascular chronic complications (OR 3.77; 95% CI, 2.51–5.78). Detailed results are presented in Table 2.

**Table 2:** Results of the univariate linear regression analysis

Variable	*p* value	OR	95% CI
Gender F/M [*n*/*n*]	0.613	0.87	(0.51; 1.47)
Age [yrs]	<0.001	1.07	(1.05; 1.1)
Diabetes duration [yrs]	<0.001	1.16	(1.12; 1.2)
HbA1c [%]	0.018	1.26	(1.04; 1.54)
Obesity [Yes/No]	0.106	1.86	(0.88; 3.97)
BMI [kg/m^2^]	0.529	1.02	(0.96; 1.08)
Peripheral neuropathy [Yes/No]	<0.001	4.72	(2.97; 7.61)
Autonomic neuropathy [Yes/No]	<0.001	7.76	(2.84; 27.25)
DKD [Yes/No]	0.001	6.28	(2.23; 22.37)
Stroke/TIA [Yes/No]	0.762	1.54	(0.06; 39.06)
CAD [Yes/No]	0.358	2.33	(0.38; 17.82)
Hypertension [Yes/No]	<0.001	2.87	(1.62; 5.2)
Atherosclerosis of the arteries of the lower extremities [Yes/No]	0.184	4.67	(0.59; 94.97)
Hypothyroidism [Yes/No]	0.307	1.25	(0.82; 1.91)
Total Cholesterol [mmol/l]	0.008	1.5	(1.12; 2.05)
LDL [mmol/l]	<0.001	1.9	(1.34; 2.75)
TG [mmol/l]	0.303	1.26	(0.81; 1.98)
Any smoking [Yes/No]	0.614	1.21	(0.57; 2.5)
Pregnancy during the follow-up [Yes/No]	0.367	1.21	(0.8; 1.83)
Any micro or macrovascular complications [Yes/No]	<0.001	3.77	(2.51; 5.78)

The following variables were included in the multivariate model: age, diabetes duration, HbA1c level, arterial hypertension, LDL level, and the presence of microvascular and macrovascular complications combined. In the multivariate logistic regression model, independent risk factors for DR remained: T1DM duration (OR 1.13; 95% CI, 1.09–1.19), HbA1c level (OR 1.41; 95% CI, 1.08–1.84), LDL level (OR 1.79; 95% CI, 1.16–2.87), and the presence of the combined non-DR micro- and macrovascular chronic complications (OR 1.86; 95% CI, 1.16–3.03). Detailed results are presented in Table 3.

**Table 3:** Results of the multivariate linear regression analysis

Variable	*P* value	OR	95% CI
Age [yrs]	0.543	1.01	(0.98; 1.05)
Diabetes duration [yrs]	<0.001	1.13	(1.09; 1.19)
HbA1c [%]	0.012	1.41	(1.08; 1.84)
Hypertension [Yes/No]	0.991	0.99	(0.45; 2.18)
LDL [mmol/l]	0.011	1.79	(1.16; 2.87)
Any micro or macrovascular complications [Yes/No]	0.011	1.86	(1.16; 3.03)

## Discussion

A century after the discovery of insulin, DR still affects thousands of patients with T1DM worldwide [20, 22–24]. In this retrospective RWD study from a highly selected cohort, we report that among T1DM patients with a long-term disease and current excellent glycemic control, treated with modern therapies and remaining under intensive diabetic counselling, the prevalence of DR, particularly at the advanced stage, was lower than previously described in other populations. We also provide evidence that well-established independent risk factors, such as duration of diabetes, glycemic control and some others, confirmed in this very selected cohort of T1DM patients to be significant.

Of note, in this study group new methods of T1DM treatment were commonly used, with almost all patients using insulin analogues and two-thirds being on personal insulin pumps. In addition, these patients remained, at least in recent years, under comprehensive, structured diabetes care, with frequent check-ups at a diabetologist’s office, multi-specialist care, and regular screening for comorbidities. The prevalence of DR of any stage in this ethnically and clinically homogeneous group was <40%, in spite of long diabetes duration. In a pilot of The Polish Diabetes Registry for Adults dated from 2006 to 2009, the prevalence of any stage DR in non-selected 1040 T1DM individuals with a mean disease duration slightly above 14 years (a period much shorter than in the current study) was almost 42% [35]. A more recent nationwide study of DR in Poland in the years 2013–2017 based on the International Classification of Diseases Codes (ICD-9 and ICD-10) and unique National Health Fund codes reported a 20.0% prevalence of DR in T1DM patients [36]. Of note, there are substantial methodological differences between this and our study, as all T1DM individuals were included without regard to the disease duration, and, moreover, the frequency of DR was likely underreported.

Among the reports from other countries, a large observational study was conducted in the USA between 2001 and 2014. This study revealed a DR rate of 20.1% in a group of patients with a median age of T1DM diagnosis 14.2 years, follow-up of 3.2 years and HbA1c level 7.6% [10]. A prospective observational analysis showed an increased frequency of DR, with subsequent years reaching almost 40% after 10 years, half of the average T1DM duration for the current study [10].

In a pilot SEARCH study that analysed data from 2009 to 2011 in the US, the presence of DR was assessed based on photographs of the retina without pupil dilation; the frequency of DR was estimated to be 17% at mean, time from diagnosis slightly <6.8 years [13]. In a large British cross-sectional analysis based on the screening program among patients with T1DM (aged > 12 years, regardless of the duration of the disease) and covering the years between 2005 and 2009, the frequency of any DR and any sight-threatening retinopathy was 56.0% and 11.2%, respectively; numbers higher than in our study, with a shorter mean T1DM duration of less 22.3 years [12].

In a report from Sweden dated back to 2008, the prevalence of DR in the T1DM population was estimated at 41.8%, PDR rate was 8.5%, and the percentage of people at risk of blindness reached 12.1% [37]. Of note, these estimates were based on large patient populations, but without taking into account the T1DM duration. Data extracted in 2014 from the Danish island of Funen showed an even higher proportion of DR – 54.3% for any DR, 16.4% for PDR - - in the T1DM patient population with a median duration of disease lasting 19.1 years [38].

In the Norwegian study, showing data from the last decade, where the studied cohort was similar to one in this study in terms of age (mean 34 yrs) and T1DM duration (mean 19 years), the DR rate was 61% and 18% for severe DR; this group, however, was characterised by an unsatisfactory metabolic control with mean HbA1c 8.2% [11]. Compared to this study, our cohort was characterised by a lower percentage of people with advanced DR changes including PDR (3.4% vs. 13%, respectively), and a lower prevalence of DME (1% vs. 8%). Our analysis showed no association between the presence of DR and the male gender, although this gender was underrepresented in the current analysis. In addition, the specific DR diagnosis in our study was based on the direct examination of the retina by an ophthalmologist, unlike in the Norwegian study where a fundus photo was used. Data from other countries on the frequency of DR among patients with long-standing T1DM treated with modern therapeutic methods are scarce.

Finally, to illustrate the differences in a much longer perspective, we should refer to the WESDR study published almost 40 years ago, where the prevalence of PDR after 15 or more years of T1DM was 20.1%, more than two times higher than severe DR in the current cohort and more than six times higher than PDR in the current cohort [9].

In summary, while the comparison of studies from different countries evaluating the prevalence of DR in T1DM seems to be challenging due to some differences in methodology and characteristics of the study groups, it is apparent that the frequency of both any DR and severe DR in this report are lower than in earlier studies.

The results of our study, with a lower than earlier reported prevalence of DR, support the individualised attitude to screening for DR in T1DM patients. The guidelines of the ADA [39], American Academy of Ophthalmology [30] and the Polish Diabetes Association [40], recommend starting screening for DR as early as 5 years after the diagnosis of T1DM. In the absence of evidence of DR after examination, and with good glycemic control, ophthalmologists and optometrists are currently advised to perform eye examinations every 1–2 years. If any degree of DR is present, a subsequent ophthalmologic examination with dilatated pupils should be repeated at least annually. If there are no signs of DR in subsequent examinations, longer intervals between follow-up examinations may be considered. We also suggest that the frequency of screening for DR should be based on the presence of other chronic diabetes complications. Screening for DR at fixed annual intervals is challenging to the health care system. To overcome this barrier, the individual attitude seems to be safe for patients and economically beneficial [41].

In this retrospective analysis, we also examined the risk factors for any stage of DR. We identified the T1DM duration and glycemic control among them. This is not surprising, as there is well-known and consistent evidence that a longer duration of T1DM and worse metabolic control as expressed by higher HbA1c levels increase the risk of development and progression of DR [8–10, 18, 24, 42]. The WESDR study reported that the severity of DR was also associated with a higher level of HbA1c [9]. The DCCT study and its follow-up EDIC clearly demonstrated that lower HbA1c levels as a result of intensive care were correlated with a later onset and the slower development of DR [43]. Our RWD study confirms this observation in a smaller but homogeneous cohort of T1DM patients using modern therapies and reaching satisfactory glycemic control. Another risk factor identified in our study was the LDL level. It was earlier shown that higher levels of cholesterol correlate with the appearance of “hard exudates” in the retina [44]. Some reports show that high LDL level was a risk factor for DR progression [45, 46]. Moreover, treatment of dyslipidemia inhibited the progression of the already diagnosed disease [20]. The FIELD study clearly showed that fenofibrate treatment in patients with T2DM reduced the progression of DR and need for laser DR treatment [47]. Our study emphasises the role of LDL cholesterol as a risk factor of DR. Unexpectedly, arterial hypertension was not an independent DR risk factor in our multivariate analysis. One of the reasons influencing the statistical analysis could have been the well-documented relationship between arterial hypertension and many non-DR micro- and macrovascular chronic complications, that were combined as a single variable in this study [48].

Of note, we also demonstrated the relationships between DR and the presence of any chronic complication. Earlier studies analysed the association between DR and individual microvascular and macrovascular complication, usually DKD [49]. In the WESDR study, proteinuria was a predictor of PDR development in patients with T1DM [50]. In the fourteen-year follow-up of the Wisconsin study, the increased risk of macular edema was associated with the presence of high proteinuria at baseline [51]. In a study including T1DM subjects with preclinical diabetic glomerulopathy lesions, a significant relationship was found between DR and morphological changes in kidneys [52]. However, there has been no earlier reports in which chronic complications would be treated jointly in association analysis with DR.

This report has some limitations. Our cohort is characterised by the over-representation of female T1DM patients with a more than four times larger proportion than males. This is a characteristic feature of the T1DM cohort of our centre [53]. Women with T1DM are attracted to our department as they have a special program dedicated to pregnancy planning and care. This includes the possibility of borrowing an insulin pump, otherwise available in Poland only from out-of-pocket payments for T1DM patients above the age of 26 yrs. The National Health Fund partially covers the costs of equipping the pump for pregnant women with diabetes. In the analysed group, over half of female patients were pregnant during the follow-up period. After the delivery, these patients usually remain in the KAOS program. However, as shown in our subgroup analysis, neither the prevalence of any DR nor the severity of DR were associated with gender in our study. Further, this was a retrospective study and we cannot definitely prove any causal relationship as the results just reflect a statistical association between the examined variables. The analysed group of patients was a relatively homogeneous cohort remaining under the care of the university center, thus, the results cannot be extrapolated to the entire population of T1DM patients in Poland. Our analyses did not take into account some important clinical variables that could potentially influence the results, such as the glycemic control and treatment of the patients before their admission to the KAOS program. We were also not able to provide a precise number of T1DM patients using the CGM systems, although our model of care included supplying pregnant women or planning pregnancy in sensors for CGM. Moreover, data on severe hypoglycemia and hyperglycemic episodes were not systematically collected for this cohort. Finally, it should be admitted that the cut-off point of 10 years for T1DM duration was arbitrary in its nature. It was well-documented that sufficient glycemic exposure was needed for the development of DR [20]. As a consequence, the first ophthalmological examination for T1DM patients is recommended 5 years after the diagnosis [39]. Of note, severe DR usually did not develop before 10 year of T1DM [15, 20] and this cut-off point was used in some earlier studies of DR [54]. Among the advantages, one should include the assessment by the same team of specialists in ophthalmology.

## Conclusions

In this highly-selected group of mostly female T1DM patients, the prevalence of both any stage of DR and severe DR were lower than previously reported from other populations. We confirmed most independent risk factors of DR from earlier studies involving cohorts with different clinical characteristics.

## Supplementary information

Supplementary Appendix1

## References

[1] Cheung N, Mitchell P, Wong TY (2010). Diabetic retinopathy. Lancet.

[2] Bourne RR, Stevens GA, White RA, Smith JL, Flaxman SR, Price H, Jonas JB, Keeffe J, Leasher J, Naidoo K, Pesudovs K, Resnikoff S, Taylor HR (2013). Causes of vision loss worldwide, 1990–2010: a systematic analysis. Lancet Glob. Health.

[3] Esteves JF, Dal Pizzol MM, Sccoco CA, Roggia MF, Milano SB, Guarienti JA, Rodrigues TC, Canani LH (2008). Cataract and type 1 diabetes mellitus. Diabetes Res Clin. Pract..

[4] Zhao D, Cho J, Kim MH, Friedman DS (2015). Guallar E. Diabetes, fasting glucose, and the risk of glaucoma: a meta-analysis. Ophthalmology.

[5] Simó R, Stehouwer CDA, Avogaro A (2020). Diabetic retinopathy: looking beyond the eyes. Diabetologia.

[6] Simó R, Bañeras J, Hernández C, Rodríguez-Palomares J, Valente F, Gutierrez L, González-Alujas T, Ferreira I, Aguadé-Bruix S, Montaner J, Seron D, Genescà J, Boixadera A, García-Arumí J, Planas A, Simó-Servat O, García-Dorado D (2019). Diabetic retinopathy as an independent predictor of subclinical cardiovascular disease: baseline results of the PRECISED study. BMJ Open Diabetes Res. Care..

[7] J. Xie, M.K. Ikram, M.F. Cotch, B. Klein, R. Varma, J.E. Shaw, R. Klein, P. Mitchell, E.L. Lamoureux, T.Y. Wong, Association of diabetic macular edema and proliferative diabetic retinopathy cardiovascular disease: a systematic review and meta-analysis. JAMA Ophthalmol. **135**(Jun 6), 586–593 (2017). PMID: 28472362; PMCID: PMC5593137 10.1001/jamaophthalmol.2017.0988 10.1001/jamaophthalmol.2017.0988 PMC5593137 28472362

[8] Yau JW, Rogers SL, Kawasaki R, Lamoureux EL, Kowalski JW, Bek T, Chen SJ, Dekker JM, Fletcher A, Grauslund J, Haffner S, Hamman RF, Ikram MK, Kayama T, Klein BE, Klein R, Krishnaiah S, Mayurasakorn K, O’Hare JP, Orchard TJ, Porta M, Rema M, Roy MS, Sharma T, Shaw J, Taylor H, Tielsch JM, Varma R, Wang JJ, Wang N, West S, Xu L, Yasuda M, Zhang X, Mitchell P, Wong TY (2012). Meta-Analysis for Eye Disease (META-EYE) Study Group. Global prevalence and major risk factors of diabetic retinopathy. Diabetes Care..

[9] R. Klein, B.E. Klein, S.E. Moss, M.D. Davis, D.L. DeMets, The Wisconsin epidemiologic study of diabetic retinopathy. III Prevalence and risk of Diabetic retinopathy when age diagnosis is 30 or more years. Arch. Ophthalmol. **102**(Apr 4), 527–532 (1984). PMID: 6367725 10.1001/archopht.1984.01040030405011 10.1001/archopht.1984.01040030405011 6367725

[10] Wang SY, Andrews CA, Herman WH, Gardner TW, Stein JD (2017). Incidence and risk factors for developing diabetic retinopathy among youths with Type 1 or Type 2 diabetes throughout the United States. Ophthalmology.

[11] Jansson RW, Hufthammer KO, Krohn J (2018). Diabetic retinopathy in type 1 diabetes patients in Western Norway. Acta Ophthalmol..

[12] Thomas RL, Dunstan FD, Luzio SD, Chowdhury SR, North RV, Hale SL, Gibbins RL, Owens DR (2015). Prevalence of diabetic retinopathy within a national diabetic retinopathy screening service. Br. J. Ophthalmol..

[13] Mayer-Davis EJ, Davis C, Saadine J, D’Agostino RB, Dabelea D, Dolan L, Garg S, Lawrence JM, Pihoker C, Rodriguez BL, Klein BE, Klein R, SEARCH for Diabetes in Youth Study Group (2012). Diabetic retinopathy in the SEARCH for diabetes in youth cohort: a pilot study. Diabet. Med..

[14] Madeira C, Lopes M, Laiginhas R, Neves J, Rosas V, Barbosa M, Carvalho D, Falcão-Reis F, Falcão M (2019). Changing trends in the prevalence of diabetic retinopathy in type 1 diabetes mellitus from 1990 to 2018: a retrospective study in a Portuguese population. Diabetes Res Clin Pract.

[15] Kytö JP, Harjutsalo V, Forsblom C, Hietala K, Summanen PA, Groop PH, FinnDiane Study Group (2011). Decline in the cumulative incidence of severe diabetic retinopathy in patients with type 1 diabetes. Diabetes Care..

[16] UK Prospective Diabetes Study Group (1998). Tight blood pressure control and risk of macrovascular and microvascular complications in type 2 diabetes: UKPDS 38. UK Prospective Diabetes Study Group. BMJ.

[17] Gallego PH, Craig ME, Hing S, Donaghue KC (2008). Role of blood pressure in development of early retinopathy in adolescents with type 1 diabetes: prospective cohort study. BMJ.

[18] Hainsworth DP, Bebu I, Aiello LP, Sivitz W, Gubitosi-Klug R, Malone J, White NH, Danis R, Wallia A, Gao X, Barkmeier AJ, Das A, Patel S, Gardner TW, Lachin JM (2019). Diabetes Control and Complications Trial (DCCT)/Epidemiology of Diabetes Interventions and Complications (EDIC) Research Group. Risk factors for retinopathy in type 1 diabetes: The DCCT/EDIC Study. Diabetes Care..

[19] Bulum T, Tomić M, Duvnjak L (2017). Total serum cholesterol increases risk for development and progression of nonproliferative retinopathy in patients with type 1 diabetes without therapeutic intervention: prospective, observational study. Arch. Med Res..

[20] Chew EY, Ambrosius WT, Davis MD, Danis RP, Gangaputra S, Greven CM, Hubbard L, Esser BA, Lovato JF, Perdue LH, Goff DC, Cushman WC, Ginsberg HN, Elam MB, Genuth S, Gerstein HC, Schubart U, Fine LJ, ACCORD Study Group; ACCORD Eye Study Group (2010). Effects of medical therapies on retinopathy progression in type 2 diabetes. N. Engl. J. Med..

[21] Harvey JN (2011). The influence of sex and puberty on the progression of diabetic nephropathy and retinopathy. Diabetologia.

[22] Diabetes Control and Complications Trial Research Grou (2000). Effect of pregnancy on microvascular complications in the diabetes control and complications trial. The Diabetes Control and Complications Trial Research Group. Diabetes Care..

[23] Estacio RO, McFarling E, Biggerstaff S, Jeffers BW, Johnson D, Schrier RW (1998). Overt albuminuria predicts diabetic retinopathy in Hispanics with NIDDM. Am. J. Kidney Dis..

[24] Nathan DM, Genuth S, Lachin J, Cleary P, Crofford O, Davis M, Rand L, Siebert C, Diabetes Control and Complications Trial Research Group (1993). The effect of intensive treatment of diabetes on the development and progression of long-term complications in insulin-dependent diabetes mellitus. N. Engl. J. Med.

[25] Intensive blood-glucose control with sulphonylureas or insulin compared with conventional treatment and risk of complications in patients with type 2 diabetes (UKPDS 33). UK Prospective Diabetes Study (UKPDS) Group. Lancet. **352**(Sep 9131), 837–853 (1998). Erratum in: Lancet 1999 Aug 14;354(9178):602. PMID: 9742976 9742976

[26] J.M. Lachin, N.H. White, D.P. Hainsworth, W. Sun, P.A. Cleary, D.M. Nathan, Diabetes Control and Complications Trial (DCCT)/Epidemiology of Diabetes Interventions and Complications (EDIC) Research Group, Effect of intensive diabetes therapy on the progression of diabetic retinopathy in patients with type 1 diabetes: 18 years of follow-up in the DCCT/EDIC. Diabetes **64**(Feb 2), 631–642 (2015). Epub 2014 Sep 9. PMID: 25204977; PMCID: PMC4303965 10.2337/db14-0930 10.2337/db14-0930 PMC4303965 25204977

[27] Šoupal J, Petruželková L, Grunberger G, Hásková A, Flekač M, Matoulek M, Mikeš O, Pelcl T, Škrha J, Horová E, Škrha J, Parkin CG, Svačina Š, Prázný M (2020). Glycemic outcomes in adults with T1D are impacted more by continuous glucose monitoring than by insulin delivery method: 3 years of follow-up from the COMISAIR study. Diabetes Care..

[28] Zabeen B, Craig ME, Virk SA, Pryke A, Chan AK, Cho YH, Benitez-Aguirre PZ, Hing S, Donaghue KC (2016). Insulin pump therapy is associated with lower rates of retinopathy and peripheral nerve abnormality. PLoS ONE.

[29] Downie E, Craig ME, Hing S, Cusumano J, Chan AK, Donaghue KC (2011). Continued reduction in the prevalence of retinopathy in adolescents with type 1 diabetes: role of insulin therapy and glycemic control. Diabetes Care..

[30] American Academy of Ophthalmology. Diabetic retinopathy preferred practise pattern 2019, P66-P147. 10.1016/j.ophtha.2019.09.025, entrance date: 5.02.2020

[31] Kidney Disease: Improving Global Outcomes (KDIGO) Diabetes Work Group (2020). KDIGO 2020 clinical practice guideline for diabetes management in chronic kidney disease. Kidney Int..

[32] Z. Reiner, A.L. Catapano, G. De Backer, I. Graham, M.R. Taskinen, O. Wiklund, S. Agewall, E. Alegria, M.J. Chapman, P. Durrington, S. Erdine, J. Halcox, R. Hobbs, J. Kjekshus, P.P. Filardi, G. Riccardi, R.F. Storey, D. Wood, European Association for Cardiovascular Prevention & Rehabilitation, ESC Committee for Practice Guidelines (CPG) 2008-2010 and 2010-2012 Committees, ESC/EAS Guidelines for the management of dyslipidaemias: the Task Force for the management of dyslipidaemias of the European Society of Cardiology (ESC) and the European Atherosclerosis Society (EAS). Eur. Heart J. **32**(Jul 14), 1769–1818 (2011). Epub 2011 Jun 28. PMID: 21712404 10.1093/eurheartj/ehr158 10.1093/eurheartj/ehr158 21712404

[33] Catapano AlbericoL, Graham Ian, De Backer Guy, Wiklund Olov, Chapman MJohn, Drexel Heinz, Hoes ArnoW, Jennings CatrionaS, Landmesser Ulf, Pedersen TerjeR, Reiner Željko, Riccardi Gabriele, Taskinen Marja-Riita, Tokgozoglu Lale, Verschuren WMMonique, Vlachopoulos Charalambos, Wood DavidA, Zamorano JoseLuis, Cooney Marie-Therese, ESC Scientific Document Group (2016). 2016 ESC/EAS guidelines for the management of dyslipidaemias. Eur. Heart J..

[34] Mach F, Baigent C, Catapano AL, Koskinas KC, Casula M, Badimon L, Chapman MJ, De Backer GG, Delgado V, Ference BA, Graham IM, Halliday A, Landmesser U, Mihaylova B, Pedersen TR, Riccardi G, Richter DJ, Sabatine MS, Taskinen MR, Tokgozoglu L, Wiklund O, ESC Scientific Document Group (2020). 2019 ESC/EAS Guidelines for the management of dyslipidaemias: lipid modification to reduce cardiovascular risk. Eur. Heart J..

[35] Witek PW, Wolkow P, Stancel-Mozwiłło J, Wojtyczek K, Sieradzki J, Malecki M (2012). The Polish diabetes registry for adults - A pilot study. Diabetologia Kliniczna.

[36] Kozioł M, Nowak MS, Udziela M, Piątkiewicz P, Grabska-Liberek I, Szaflik JP (2020). First nation-wide study of diabetic retinopathy in Poland in the years 2013–2017. Acta Diabetol..

[37] Heintz E, Wiréhn AB, Peebo BB, Rosenqvist U, Levin LA (2010). Prevalence and healthcare costs of diabetic retinopathy: a population-based register study in Sweden. Diabetologia.

[38] Larsen MB, Henriksen JE, Grauslund J, Peto T (2017). Prevalence and risk factors for diabetic retinopathy in 17 152 patients from the island of Funen, Denmark. Acta Ophthalmol..

[39] American Diabetes Association. 11. Microvascular complications and foot care: standards of medical care in diabetes-2021. Diabetes Care. 44 (Jan Suppl 1), S151–S167 (2021). 10.2337/dc21-S011. PMID: 33298422 10.2337/dc21-S011 33298422

[40] 2020 Guidelines on the management of diabetic patients (2020). A position of Diabetes Poland. Clin. Diabetol..

[41] Nathan DM, Bebu I, Hainsworth D, Klein R, Tamborlane W, Lorenzi G, Gubitosi-Klug R, Lachin JM, DCCT/EDIC Research Group (2017). Frequency of evidence-based screening for retinopathy in type 1 diabetes. N. Engl. J. Med..

[42] Klein R, Klein BE, Moss SE, Davis MD, DeMets DL (1988). Glycosylated hemoglobin predicts the incidence and progression of diabetic retinopathy. JAMA.

[43] L.P. Aiello,DCCT/EDIC Research Group, Diabetic retinopathy and other ocular findings in the diabetes control and complications trial/epidemiology of diabetes interventions and complications study. Diabetes Care. **37**(1), 17–23 (2014). PMID: 24356593; PMCID: PMC3867989 10.2337/dc13-2251 10.2337/dc13-2251 PMC3867989 24356593

[44] Chew EY, Klein ML, Ferris FL, Remaley NA, Murphy RP, Chantry K, Hoogwerf BJ, Miller D (1996). Association of elevated serum lipid levels with retinal hard exudate in diabetic retinopathy. Early Treatment Diabetic Retinopathy Study (ETDRS) Report 22. Arch. Ophthalmol..

[45] M.F. Lopes-Virella, N.L. Baker, K.J. Hunt, T.J. Lyons, A.J. Jenkins, G. Virella,DCCT/EDIC Study Group, High concentrations of AGE-LDL and oxidized LDL in circulating immune complexes are associated with progression of retinopathy in type 1 diabetes. Diabetes Care. **35**(Jun 6), 1333–1340 (2012). Epub 2012 Apr 17. PMID: 22511260; PMCID: PMC3357232 10.2337/dc11-2040 10.2337/dc11-2040 PMC3357232 22511260

[46] Y. Zhou, C. Wang, K. Shi, X. Yin, Relationship between dyslipidemia and diabetic retinopathy: a systematic review and meta-analysis. Med. (Baltim.). **97**(Sep 36), e12283 (2018). 10.1097/MD.0000000000012283. PMID: 30200172; PMCID: PMC6133445 10.1097/MD.0000000000012283 PMC6133445 30200172

[47] Keech AC, Mitchell P, Summanen PA, O’Day J, Davis TM, Moffitt MS, Taskinen MR, Simes RJ, Tse D, Williamson E, Merrifield A, Laatikainen LT, d’Emden MC, Crimet DC, O’Connell RL, Colman PG (2007). FIELD study investigators. Effect of fenofibrate on the need for laser treatment for diabetic retinopathy (FIELD study): a randomised controlled trial. Lancet.

[48] Yamazaki D, Hitomi H, Nishiyama A (2018). Hypertension with diabetes mellitus complications. Hypertens. Res..

[49] Pearce I, Simó R, Lövestam-Adrian M, Wong DT, Evans M (2019). Association between diabetic eye disease and other complications of diabetes: Implications for care. A systematic review. Diabetes Obes. Metab..

[50] Klein R, Knudtson MD, Lee KE, Gangnon R, Klein BE (2008). The Wisconsin Epidemiologic Study of Diabetic Retinopathy: XXII the twenty-five-year progression of retinopathy in persons with type 1 diabetes. Ophthalmology.

[51] Klein R, Klein BE, Moss SE, Cruickshanks KJ (1998). The Wisconsin Epidemiologic Study of Diabetic Retinopathy: XVII. The 14-year incidence and progression of diabetic retinopathy and associated risk factors in type 1 diabetes. Ophthalmology.

[52] Klein R, Zinman B, Gardiner R, Suissa S, Donnelly SM, Sinaiko AR, Kramer MS, Goodyer P, Moss SE, Strand T, Mauer M (2005). Renin-Angiotensin System Study. The relationship of diabetic retinopathy to preclinical diabetic glomerulopathy lesions in type 1 diabetic patients: the Renin-Angiotensin System Study. Diabetes.

[53] Matejko B, Skupien J, Mrozińska S, Grzanka M, Cyganek K, Kiec-Wilk B, Malecki MT, Klupa T (2015). Factors associated with glycemic control in adult type 1 diabetes patients treated with insulin pump therapy. Endocrine.

[54] Henricsson M, Nyström L, Blohmé G, Ostman J, Kullberg C, Svensson M, Schölin A, Arnqvist HJ, Björk E, Bolinder J, Eriksson JW, Sundkvist G (2003). The incidence of retinopathy 10 years after diagnosis in young adult people with diabetes: results from the nationwide population-based Diabetes Incidence Study in Sweden (DISS). Diabetes Care.

