# Supplementary material for: Low prevalence of diabetic retinopathy in patients with long-term type 1 diabetes and current good glycemic control - one-center retrospective assessment
Source: Endocrine. 2021 Sep 20;75(2):427–36. doi: 10.1007/s12020-021-02871-2 (PMC8816752; doi:10.1007/s12020-021-02871-2)
Supplement: Supplementary file 1 — Supplementary Appendix1 [file 12020_2021_2871_MOESM1_ESM.docx]

Appendix 1

Use of concomitant medications

| **Group of medications** | **N** | **%** | **N in DR group** | **% in DR group** | **N in non-DR group** | **%in non-DR group** |
| --- | --- | --- | --- | --- | --- | --- |
| ACE-I | 49 | 12.8 | 31 | 20.7 | 18 | 7.7 |
| Beta-blockers | 31 | 8.1 | 24 | 16.0 | 7 | 3.0 |
| Ca-blockers | 19 | 4.9 | 13 | 8.7 | 6 | 2.6 |
| Diuretics | 18 | 4.7 | 12 | 8.0 | 6 | 2.6 |
| Alpha 1- blockers | 5 | 1.3 | 3 | 2.0 | 2 | 0.9 |
| Statins | 50 | 13.0 | 30 | 20.0 | 20 | 8.5 |
| Fibrate | 1 | 0.3 | 0 | 0 | 1 | 0.5 |
| Ezetimibe | 2 | 0.5 | 0 | 0 | 2 | 0.9 |
| Psychiatric medications | 27 | 7.0 | 12 | 8.0 | 15 | 6.4 |
| ASA | 22 | 5.7 | 12 | 8.0 | 10 | 4.3 |
| Vitamin D3 | 143 | 37.2 | 62 | 41.3 | 81 | 34.6 |
| Multivitamin supplements | 112 | 29.2 | 48 | 32 | 64 | 27.4 |
| Alpha-liphoic acid | 15 | 3.9 | 12 | 8.0 | 3 | 1.3 |
| Thiamin | 8 | 2.1 | 6 | 4.0 | 2 | 0.9 |
